# Exploring regulatory flexibility to create novel incentives to optimize drug discovery

**DOI:** 10.3389/fmed.2024.1379966

**Published:** 2024-05-13

**Authors:** Jacqueline A. Sullivan, E. Richard Gold

**Affiliations:** ^1^Department of Philosophy, Rotman Institute of Philosophy and Western Institute for Neuroscience, Western University, London, ON, Canada; ^2^Faculty of Law and Faculty of Medicine and Health Sciences, McGill University, Montreal, QC, Canada

**Keywords:** collaboration, drug development, incentives, open science, patents, regulatory exclusivity

## Abstract

Efforts by governments, firms, and patients to deliver pioneering drugs for critical health needs face a challenge of diminishing efficiency in developing those medicines. While multi-sectoral collaborations involving firms, researchers, patients, and policymakers are widely recognized as crucial for countering this decline, existing incentives to engage in drug development predominantly target drug manufacturers and thereby do little to stimulate collaborative innovation. In this mini review, we consider the unexplored potential within pharmaceutical regulations to create novel incentives to encourage a diverse set of actors from the public and private spheres to engage in the kind of collaborative knowledge exchange requisite for fostering enhanced innovation in early drug development.

## Introduction

While governments (1), firms (2), and patients (3) seek to deliver new medicines for pressing health needs—particularly first-in-indication drugs or first effective drugs for diseases for which there is no good therapy—they face a problem of declining efficiency in developing those medicines (4). The result is not only decreasing investigation into higher risk research but also lack of affordability and access (5). For example, firms are decreasing investments in neurodegenerative diseases despite estimates that dementia will affect 100 million people by 2050 (6) and not investing in antimicrobials despite predictions that 10 million people will die annually from lack of these drugs by 2050 (7). While there are between 263 and 446 million people suffering from rare diseases, 95% of these diseases lack even one treatment (8). This is despite the fact that over half of new drug approvals in both the United States and Europe are for orphan diseases, partially because firms are so adapt at strategic use of the orphan drug designation to fund large, even blockbuster, drugs (9–11) and because the largest proportion of orphan drugs are in oncology even though 80% of rare diseases are genetic in origin (12).

Partially in response to this productivity decline, firms, researchers, patients, and policy makers have moved toward a model of multi-sector and interdisciplinary collaborations to advance drug discovery (13–15). Such collaborations have been referred to as “Open Science Partnerships” (16), and they have been described as having different archetypes (17). For these collaborations to bear fruit, however, “there need to be the appropriate incentives in place, the engagement of many different stakeholders including patients and regulators but most of all people with the right expertise and enthusiasm to fully realize the value of new partnering models for human health” (14). While incentives exist in many settings—academic promotion, health care outcomes, advanced market commitments—drug regulations provide an underinvestigated opportunity to create them (18, 19). In this mini review, we explore this topic.

Collaborations between firms, university researchers, patients, philanthropies, and governments accelerate drug development by relying on the differential expertise of these actors, increasing the sharing of knowledge between them, building trust in science and its outcomes, and avoiding duplication (5, 16, 20–24). Because of the importance of health and safety in making drugs available to patients, the relationship between these actors is mediated, in part, through drug regulation and the agencies—such as the Food and Drug Administration (FDA) and the European Medicines Agency—that enforce them. As Eisenberg (18) notes, however, these regulations provide both incentives and disincentives to develop drugs: “[T] he FDA’s core function of reviewing data from clinical trials to determine the safety and efficacy of drugs prior to market approval may be understood as a means of promoting costly investments in a particular form of R&D rather than simply as a means of protecting patients from untoward risks of harm.” Acknowledging the role of collaborations in contemporary pharmaceutical research, here we examine how drug regulations, and the agencies that are responsible for them, create or could create incentives and disincentives for the various actors engaged in drug regulation to render them more efficient.

We begin, in Section 1, with a brief overview of the drug development process before moving on, in Section 2, to consider current incentives for actors to invest time and money in drug development. We note that these incentives are geared primarily at the private sector and are not well designed to encourage multi-sectoral participation in collaborations. We end, in Section 3, by considering the question of whether there is flexibility in the current system to introduce incentives for the plurality of actors who are actively involved in early drug development to work collaboratively to promote innovation.

## Drug development

**Figure 1:**
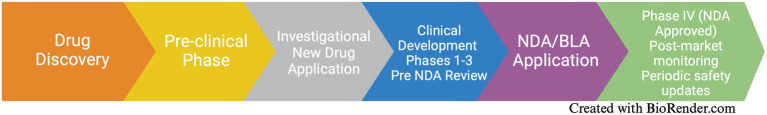
The drug development process and regulatory framework.

Drug development is usually described as a linear process with discrete scientific stages corresponding to safety and efficacy benchmarks set by regulations and the administrative bodies that apply them, such as the FDA [Figure 1; e.g., (14)]. After identifying potential compounds that could serve as drug candidates, researchers determine, in the pre-clinical stage, the toxicity and efficacy of these compounds to treat a specific disease *in vitro* (e.g., cell cultures, organoids) and *in vivo* (e.g., animal models of disease) in industrial and/or academic settings. If a drug passes these preclinical tests, the drug’s sponsor submits an Investigational New Drug Application (INDA) to the FDA to move to the next stage of assessing safety and efficacy in human subjects. Following the FDA’s approval of the INDA, the sponsor proceeds with clinical trials of the drug in small groups of human volunteers (25). These Phase I trials (20–80 subjects) provide a preliminary understanding of a drug’s safety, dosage, and identify potential side effects. If the drug meets Phase I safety benchmarks, the sponsor seeks approval to enter into larger Phase II clinical trials (100–300 participants) to more fully evaluate the drug’s safety and efficacy. If the drug passes that phase, the sponsor proceeds to Phase III clinical trials, which evaluate efficacy and side effects compared to currently available treatments for the disease. These studies involve many participants [1,000–3,000; with the exception of drugs for rare diseases, which involve smaller participant pools and sometimes combine Phases II&III clinical trials (26–28)]. If a drug passes Phase III, the drug manufacturer files a New Drug Application (NDA) with the FDA to seek approval to market the drug to patients. The FDA may require additional studies of the drug before approval. Once approved, the drug sponsor may conduct Phase IV clinical trials involving post-market monitoring of the drug,^1^ although up to a fifth of these trials are for pure marketing purposes (29).

The cost of meeting regulatory benchmarks at each stage of drug development increases as sponsors must enroll more subjects and more representatives from a pharmaceutical company (including administrators, investigators, clinicians, etc.) become involved. Risks of failure also increase at each stage, and failure may occur after a firm already has made a major financial investment in a drug. The only exceptions to these norms are cases of rare diseases that use much smaller patient cohorts, but cost savings from smaller trials have not incentivized large firms to invest in drug development for rare diseases. Given the high costs of drug development and an uncertain reward in the market, it is a high-risk endeavor.

## The narrow scope of current incentives

Intellectual property protection in the form of patents granted by national or regional patent offices, such as the United States Patent and Trademark Office or the European Patent Office, have served as the central incentive for private sector investment in drug development (30). These patents last 20 years from the date of filing and, depending on jurisdiction, up to five additional years to compensate for regulatory delays. This reliance on patents has shaped the direction of pharmaceutical innovation toward lower-risk research, such as second or third in class (“me-too”) drugs (31), and away from diseases where the biology is complex, such as antibiotics (32). From the perspective of large firms, the financial benefits resulting from investing in breakthrough science, especially in small or uncertain markets, is in many cases insufficient to justify the costs of investment.

Patents are not, of course, the only inducement for drug companies to take these risks. A number of other incentives for drug development exist comprising both push and pull mechanisms. Push incentives reduce research and development costs to drug sponsors. These include direct subsidies to firms and research and development tax credits, and indirect subsidies, such as investments in infrastructure and academic research grants. Pull incentives, in contrast, increase market reward. Beyond patents, these include data exclusivity, other niche exclusivities, and priority reviews. We illustrate these incentives by using the United States as an example, but similar incentives exist in other jurisdictions, such as the European Union.

The Orphan Drug Designation (ODD) is an example of a combination of push and pull incentives. It applies to drugs to treat diseases—orphan drugs (OD)—that affect fewer than 200,000 people in the United States (and a roughly *pro rata* number of people in other jurisdictions) by providing drug manufacturers with push incentives such as tax credits for certain research expenses, exemption from certain FDA fees (e.g., waivers of marketing applications fees), and research grants to subsidize clinical research costs. The ODD also includes a pull incentive, namely a 7-year market exclusivity period during which the FDA cannot approve another application for that drug for that indication (with some exceptions).

The Accelerated or Critical Path Initiative saves research and development costs and time by allowing firms to use biomarkers as surrogate endpoints for drugs for rare diseases [yet this is not without problems; (33)]. The Research and Experimentation Tax Credit, another push incentive, offers a large subsidy on incremental expenditures for experimental operations relative to a firm’s baseline level of expenditure (non-specific market).

Three Priority Review Voucher Programs (Rare Pediatric Diseases, Tropical Diseases, and Material Threat Medical Countermeasure) provide firms that develop qualifying drugs with a voucher that may be used to expedite the review of another drug, presumably one with a larger market, that is in the firm’s pipeline. Firms can either use the voucher themselves or to sell it to another firm, thus monetizing the vouchers. The latter option would be particularly attractive to small firms that may not have another drug in the pipeline. The Pediatric Exclusivity Provision (1997) allows drug companies to have 6 additional months of exclusivity for drugs that are tested in children. The Generating Antibiotic Incentives Now (GAIN) Act of 2012 allows drug manufacturers five additional years of exclusivity for certain antibiotics. Additional pull incentives include Priority Review, Fast-Track Designation, Accelerated Approval and Breakthrough Therapy Designation Pathways.

These push and pull incentives have been insufficient, on their own, to stimulate the level of investments needed to develop first-in-indication and first effective for an indication drugs and to respond to unmet health needs. For example, while the ODD has been successful for rare diseases within its purview, some claim many ODs developed are not affordable (34) and that the incentive has served to decrease interest in developing drugs for other diseases equally worthy of consideration. A performance audit of the Priority Review Voucher Program suggests that although drug sponsors, researchers, and stakeholders value it, the program tends to serve as incentive for developing me-too drugs rather than novel treatments (35).

Failure of current incentives to stimulate sufficient levels of first-in-indication and first effective for an indication drug development calls for an examination of alternative incentive mechanisms. These are not in short supply. One suggestion is, for example, to offer pharmaceutical companies tax credits to a percentage of research and development costs or to provide them with federal subsidies to engage in research and development on novel drugs (35). Other proposals seek to encourage firms to run clinical trials with individuals that are more representative of the population’s diversity. Some suggest, for example, that governments develop financial incentives (e.g., tax credits) for manufacturers to increase diversity in clinical trials in line with the Food & Drug Omnibus Reform Act of 2022 [FDORA; e.g., (36)]. An alternative suggestion for boosting diversity in clinical trials is for the government to offer sponsors that have diversity plans in place Fast-Track Designation, Priority Review, Priority Review Vouchers, or Pediatric Exclusivity (37).

The problem with all of these incentives is their exclusive focus on the financial interests of pharmaceutical firms, based on the assumption that drug sponsors alone hold the keys to the types of health innovation that patients and health systems desire. This narrow focus is unlikely to solve the problem of increasing investments into first-in-indication drugs or increasing the productivity of drug innovation. After all, firms will naturally orient themselves to investments in areas offering higher payoffs and lower risks. To gain real efficiencies, however, the drug discovery process needs to focus on *how* research is done and by *whom*.

To address declining efficiency, incentives need to be created that ease knowledge exchange, reduce barriers to knowledge production, and encourage contributions by more actors. Beyond pharmaceutical firms, there are many other actors: non-pharmaceutical firms (e.g., involved with equipment or artificial intelligence), philanthropies (including large philanthropies such as the Bill and Melinda Gates Foundation, the Chan Zuckerberg Initiatives, the Aligning Science Against Parkinson’s Initiative, and the Michael J. Fox Foundation), venture capital, angel investors, patient organizations (e.g., disease-specific patient organizations), community organizations (representing, for example, ethnic or racial minorities), academic researchers, government researchers, intergovernmental organizations (e.g., the World Health Organization, the Organization for Economic Co-operation and Development), and more. These actors bring knowledge, tools, genetic material, probes, molecules, assays, processes, coordination, financing, expertise, and commitment that, when appropriately managed, have the potential to render the drug discovery process more efficient (16).

To achieve productivity gains, we need to focus on rendering the flow of knowledge, data, and materials between actors more efficient. Think, for example, of moving data and materials from an academic lab to an industry partner in the so-called “valley of death” between “finding a promising new agent and demonstrating its safety and efficacy in humans” (38). Financial incentives for pharmaceutical firms may encourage them to seek out such transfers but we also need to find incentives for researchers and research institutions to share. The prospect of money at some future point may help, but researchers generally have more immediate concerns such as maintaining credibility, attracting and retaining junior staff, obtaining grants and awards, and reputation. Universities care about meeting short term metrics, pleasing donors and funders, rising in university rankings, and pleasing government sponsors. If we focus narrowly on economic incentives, a set of mismatched incentives is the likely result.

Beyond the single lab and isolated university lie other coordination problems. Researchers with different expertise work in silos, experimental approaches are not standardized, results are too often not reproducible, and incentives work against collaboration (e.g., promotion criteria, authorship, etc.) (39). Universities compete with each other for researchers, students, funding, and increasing the number of patents (regardless of value) with little care for actually maximizing innovation (40) or social return.

If we were to develop an understanding of the goals of the various actors who contribute in a significant manner to drug development, we could identify novel or modified incentives that we can introduce into the drug discovery system. While these incentives may be found in a variety of settings and through diverse mechanisms, here we focus on the drug regulatory system. As indicated above, this system already incorporates numerous targeted incentives—priority, data exclusivity, vouchers, etc.—yet has been underexplored as a source of incentives for actors other than sponsors.

## Actors, regulatory flexibility and the potential for novel incentives

Given the variety of actors involved, the incentives net must be cast wide. To identify potential incentives, one needs to first develop an understanding of the preferences of the different actors and the kinds of incentives to which they may be responsive based on a literature review. A variety of qualitative approaches (e.g., ethnography, surveys, semi-structured interviews) can be used to probe actor beliefs and values: from the patients who donate tissue, DNA, and blood samples, to academic research scientists involved in preclinical research, to representatives of industry seeking to develop and market a new product. An economist looking at the results can build a utility function for each action, lawyers can construct an effective governance regime to address these understandings, and entrepreneurs can develop business plans. Indeed, we have previously undertaken some of this research (41).

Consider, for example, the views of academic scientists involved in drug development. These scientists include students, early career researchers (e.g., postdoctoral research fellows and assistant professors), mid-career and senior researchers.

Students need funding to complete their studies and they need experience. Given the scarcity of academic positions, most will work in the private sector and so would value previous exposure to firms.

Early career scientists who have secured tenure-track professorships seek start-up funding for their laboratories to buy equipment and materials, to recruit qualified personnel including postdoctoral researchers, students, and research and support staff. Progression in the profession (e.g., securing tenure), requires continued financial support through successful grant applications to fund their labs and programs of research, produce publications, travel to present research at academic conferences, and building their research networks and reputations.

Senior scientists, in addition to maintaining the same level of research they have historically, seek to maintain and expand the store of data and tools that underlies their research. Because they also care about legacy, they further aim to assist junior researchers to use those data and tools and build on them. They want their students and juniors to be successful by publishing in respected journals, obtaining research funds, and networking with those inside and outside academia to facilitate job progression and reputation.

For all scientists, the quality of their research matters. In the wake of the so-called “replication crisis in science,” funders and the research community have worked to make the research process more open and transparent to facilitate replication experiments and to root out error and fraud. This has an impact on the kinds of financial resources that researchers require to uphold standards of openness and transparency. Given the complexity of the diseases for which novel drugs are sought, not only is replication important, but so is collaboration across researchers [e.g., (42)]. For example, researchers conducting *in vitro* studies need to share with those conducting *in vivo* studies and at multiple different sites. To accomplish this, they must standardize and document techniques, ensuring that the collaboration produces replicable data and high-quality data sets. This leads, in turn, to having to cover expenses for technical personal to set-up and manage databases and data repositories for the (often) high costs of publishing in open access journals. Funding for sustainability of these databases and continuity of governance are also desirable, as is a credit system in science that encourages collaboration in ways that allow individuals to contribute to the science machine while getting credit for their individual contributions.

This openness and transparency runs straight into, however, the interests of pharmaceutical companies in keeping data about promising compounds private through patents and trade secrets. It is thus an interesting question in the current climate of drug development whether partnerships between private companies and public organizations can be open and what the conditions of that openness must be to spur all actors to coordinate and share. There are some examples of successful efforts; it would be worthwhile to understand what features allowed them to be successful and, where they failed, to understand why. It is a waste of both private and public resources to invest money in drug discovery and preclinical research if that investment never leaves the bench.

With this broader perspective of actor needs, we return to the question of whether the drug regulatory system can be made to offer incentives not only to industry but to all actors to efficiently collaborate on drug development. We can consider responding, for example, to researcher needs for access to high-quality data by increasing disclosure requirements during clinical studies and, while preventing competitors from using the same data for regulatory purposes, require them to disclose more information about drugs going through the regulatory system. Regulations could also encourage firms to enter into partnerships with a broad range of actors without compromising disclosure by extending the term of data protection provided that the sponsor discloses all data and refrains from imposing patent restrictions on subsequent uses of that data to develop other drugs.

These are just examples of the type of incentives that we can build into the regulatory system beyond vouchers, priorities, and exclusivities aimed at sponsoring firms. This system is a good place to start without forgetting that incentives exist elsewhere, such as those managed by granting agencies, university promotion and tenure committee, and philanthropies.

## Discussion

Addressing declining efficiencies in drug discovery requires incentives aimed at more than one participant: the pharmaceutical firm. We can render the process more productive by eliminating duplication (by sharing data and outcomes), reducing barriers to access expertise (by facilitating exchange between academics and industry), by coordinating efforts, and by seeking monetary and in-kind investments by a broader set of actors. Experiments with such efforts are underway (43). The Structural Genomics Consortium, the Open Discovery Innovation Network, the Montreal Neurological Hospital-Institute, and the Translational Research Initiative to De-Risk Neuro-Therapeutics (TRIDENT) all seek to advance drug discovery by bringing public and private actors together in which data and outputs are shared and not patented, and in which patients are the clear focus.

Lest one think that it is not possible to align so many actors at once, consider the great advance in open access publications and data over the last two decades. In that time, through the concerted efforts of philanthropies, granting councils, and researchers, the scientific community has moved from a system of restricted access to scientific publications to a strong norm of open access and data sharing (44). The same can be done for drug discovery incentives.

## Author contributions

JS: Writing – original draft, Writing – review & editing. EG: Writing – original draft, Writing – review & editing.

## Footnotes

^1^https://www.nih.gov/health-information/nih-clinical-research-trials
